# Dietary Sodium Butyrate Supplementation Enhances Silkworm Silk Yield by Simultaneously Promoting Larval Growth and Silk Gland Development

**DOI:** 10.3390/insects16080761

**Published:** 2025-07-24

**Authors:** Xiaoxiao Ren, Xingjiang He, Zhanfeng Ye, Zhuo Qing, Wanjun Yang, Chaobin Luo, Dan Xing

**Affiliations:** 1 Institute of Sericulture, Guizhou Academy of Agricultural Sciences, Guiyang 550006, China; 15025140962@126.com (X.R.); yezhanfeng@yeah.net (Z.Y.); qingzhuo2179@163.com (Z.Q.); gzcysywj@163.com (W.Y.); 2 Institute of Integrated Agriculture Development, Guizhou Academy of Agricultural Sciences, Guiyang 550006, China; 13668732861@163.com

**Keywords:** silkworm, sodium butyrate, silk yield, body growth, silk gland development

## Abstract

Silk yield enhancement in sericulture has plateaued. Sodium butyrate (NaB) is known to improve production performance in livestock and poultry, yet its effects on silkworm silk yield remain uncharacterized. Here, we evaluated the impact of dietary NaB supplementation on silkworm growth, silk gland development, and cocoon output. Mulberry leaves were immersed in NaB solutions at concentrations of 0, 2.5, 5, 10, 20, 40, or 80 mM, and subsequently provided as feed to third- to fifth-instar larvae. Among these, 10 mM NaB treatment most effectively promoted larval and pupal weight gain and increased food intake. Phenotypic and economic trait analyses revealed that 10 mM NaB treatment significantly enlarged the silk gland and boosted overall silk yield. Mechanistically, NaB enhanced body growth by increasing feeding intake and influencing the juvenile hormone and ecdysteroid signaling pathways; moreover, it promoted DNA replication in silk gland cells, thereby influencing silk gland development. Taken together, our findings demonstrate that dietary supplementation with an appropriate concentration of NaB concurrently enhances body growth and silk gland development, leading to higher silk production, and underscore the potential of short-chain fatty acid salts in advancing sericulture.

## Simple Summary

Silkworm (*Bombyx mori*) silk possesses significant economic value, however, silk yield has reached a bottleneck, limiting the sustainable development of sericulture. Exogenous dietary supplements can positively influence silk production, yet highly effective additives remain to be discovered. Sodium butyrate is an important nutritional additive in livestock and poultry, renowned for its capacity to improve production performance, but its impact on silkworm silk yield has not been investigated. In this study, we identified the optimal dietary sodium butyrate concentration for silkworm larvae during the third to fifth instars and elucidated its effects on larval growth, silk gland development, and silk yield, offering new insights into strategies for enhancing silk production.

## 1. Introduction

Silkworms (*Bombyx mori*) derive their primary economic value from their silk [1]. Silk consists of sericin proteins that enwrap fibroin proteins, which are synthesized in the middle and posterior silk glands (MSG and PSG, respectively) and secreted into the glandular lumen for storage [2,3]. Fibroin, as a natural fiber, exhibits excellent mechanical strength and biocompatibility, making it widely used in textiles and biomedical applications [4]. Sericin, owing to its bioactivity, low immunogenicity, and biodegradability, has found applications in cosmetics, cell culture, tissue engineering, and drug delivery [5].

Silk yield is a key economic trait in sericulture, however, improvements in yield have plateaued [6]. Promoting body growth and silk gland development remains a critical strategy to overcome this bottleneck [7]. As an oligophagous insect, silkworm growth, development, and silk production heavily depend on the nutritional quality of their diet (mulberry leaves or artificial feed) [8]. Previous studies have shown that dietary supplementation of specific nutrients can enhance larval performance and silk output [9]. For instance, Alfazairy et al. (2024) reported that supplementing mulberry leaves with *Lepidium sativum* L. seed extract increased pupal weight and cocoon shell weight [10], while Lattala et al. (2014) demonstrated that mulberry leaves coated with spermidine solutions at 25 μM and 50 μM, when fed to fifth-instar silkworm larvae, significantly increased both silk gland mass and cocoon shell weight [11]. Despite these advances, the identification of dietary additives capable of further breaking through the silk-yield plateau remains an open challenge.

Butyrate, a short-chain fatty acid produced by gut microbial fermentation of dietary fiber, serves as a primary energy substrate for intestinal epithelial cells [12,13]. Exogenous butyrate supplements—most notably sodium butyrate (NaB)—have demonstrated considerable potential for enhancing growth performance and production efficiency in livestock and poultry [14,15]. For example, inclusion of 0.02% NaB in the diet of *Pelodiscus sinensis* significantly increased weight-gain rate over a 60-day feeding period [16]. In weaned piglets, dietary supplementation with 500 ppm or 1000 ppm coated sodium butyrate (CSB) for 35 days markedly improved average daily gain (ADG) [17]. Likewise, laying ducks receiving 0.25 g/kg CSB in their basal diet exhibited a significant elevation in laying rate at 53–56 weeks of age [18], and 800 mg/kg CSB in the diet of 51-week-old laying hens enhanced yolk color and egg weight [19]. Consequently, NaB is regarded as one of the most promising nutritional additives in modern animal production. However, its effects on silkworm growth, development, and silk yield have not been explored.

In this study, we investigated the impact of dietary NaB supplementation on silkworm body growth, silk gland development, and silk production. By providing third- to fifth-instar larvae with mulberry leaves soaked in 2.5, 5, 10, 20, 40, or 80 mM NaB, we found that a 10 mM NaB treatment significantly enhanced both larval and pupal weight gain and increased the feeding intake of fifth-instar larvae. Phenotypic analysis of the silk glands revealed that this concentration markedly enlarged the glands and substantially increased cocoon silk yield. These results provide the first experimental evidence that dietary NaB can concurrently enhance silkworm growth and silk gland development, thereby potentially overcoming current bottlenecks in silk production. Moreover, our findings shed light on the broader role of short-chain fatty acid salts in regulating lepidopteran development and suggest a novel dietary strategy for boosting sericultural output.

## 2. Materials and Methods

### 2.1. Experimental Insect Material

The silkworm strain “Qingsong” was kindly provided by the Sericulture Research Institute of Guizhou Academy of Agricultural Sciences (Guiyang, China). Mulberry variety “Nongsang-14” was sourced from the institute’s mulberry germplasm garden. Eggs were incubated and, upon hatching, larvae were reared on fresh mulberry leaves in an environmental chamber set to 25 ± 1 °C, 75 ± 5% relative humidity, and a 12 h light: 12 h dark photoperiod.

### 2.2. Assessment of NaB on Silkworm Growth

NaB was purchased from Beijing Solarbio Science & Technology Co., Ltd. (Beijing, China). Stock solutions of 2.5, 5, 10, 20, 40 and 80 mM NaB were prepared in ultrapure water, stored at 4 °C, and refreshed daily. Fresh mulberry leaves were immersed in the appropriate NaB solution for 15 min, allowed to drain, then offered to the larvae.

To evaluate the effects of varying NaB concentrations on silkworm larval growth, on the first day of the third instar (designated L3D1), larvae were randomly assigned to seven groups: a control group (leaves soaked in ultrapure water) and six NaB treatments (2.5, 5, 10, 20, 40, and 80 mM; hereafter referred to as NaB2.5–NaB80). Specifically, each group comprised 90 larvae (3 replicates of 30 larvae each). Larvae were fed three times daily until the end of the fifth instar, when feeding ceased. At the onset of the wandering stage (defined by cessation of feeding, body translucency, and lateral body movements as larvae began seeking cocooning sites), six larvae per replicate (total *n* = 18) were randomly selected and weighed. At 7 days after cocooning, pupae were sexed and individual weights of female and male pupae were recorded.

To assess the temporal effects of 10 mM NaB, L3D1 larvae were assigned to two groups: Control (larvae fed mulberry leaves soaked in ultrapure water) and NaB10 (larvae fed leaves soaked in 10 mM NaB solution). Each group comprised 1000 larvae, organized into five replicates of 200 larvae each. From L3D1 onward, 30 larvae per replicate were randomly sampled at the beginning of each day for body weight measurement. Mulberry leaf intake by larvae from L5D1 to L5D7 was measured: before each daily feeding, the mass of supplied leaves was recorded; after a 24-h feeding period, the residual leaf mass was weighed (3 replicates of 30 larvae each). Individual consumption was calculated as:

Leaf intake per larva = Mass supplied − Mass remaining.

To assess the impact of 10 mM NaB on the developmental timing of fifth-instar larvae, beginning at L5D7, wandering-stage larvae in both Control and NaB10 groups were quantified (three replicates of 30 larvae each). The proportion was calculated as:

[(number of wandering larvae/total number of larvae) × 100%].

### 2.3. Silk Gland Phenotype Observation and Measurement

At L5D1, L5D3, L5D5, and L5D7, ten larvae from each of the Control and NaB10 groups were randomly selected for silk gland (SG) dissection. SGs were rinsed thrice in phosphate-buffered saline (PBS, pH 7.4), transferred to sterile Petri dishes, and blotted dry. Subsequently, whole SG weight was measured using an analytical balance (Sartorius, Göttingen, Germany), with vertical hanging length recorded (*n* = 10). After separating middle (MSG) and posterior (PSG) silk glands, their lengths were measured and individually weighed (*n* = 10). For histological analysis, PSGs from L5D7 larvae of both groups were fixed overnight at 4 °C in 4% paraformaldehyde (Servicebio, Wuhan, China), embedded in paraffin, and sectioned at 10 μm thickness. Sections were deparaffinized in xylene substitute (Servicebio, China), rehydrated through graded absolute ethanol (Sinopharm Chemical Reagent Co., Ltd., Shanghai, China) to distilled water, and then stained with DAPI (Servicebio, China) for 10 min. After three washes in PBS (5 min each) with gentle agitation, slides were mounted with an anti-fade reagent (Servicebio, China) and imaged under a fluorescence microscope (Nikon, Tokyo, Japan) using 360 nm excitation.

### 2.4. DNA Content Determination

PSGs were collected from L5D7 larvae in both Control and NaB10 groups, pooling three glands per biological replicate (*n* = 3 per group). Tissues were flash-frozen in liquid nitrogen and ground to a fine powder. Genomic DNA was extracted using the DNeasy Blood & Tissue Kit (Qiagen, Hilden, Germany) following the manufacturer’s protocol. DNA concentrations were measured at 260 nm on a NanoDrop 2000c spectrophotometer (Thermo Scientific, Waltham, MA, USA).

### 2.5. Quantitative Real-Time PCR (qRT-PCR)

Whole larvae and PSGs were sampled from L5D3 of both Control and NaB10 groups, pooling three larvae or three PSGs per biological replicate. Samples were flash-frozen, ground in liquid nitrogen, and total RNA extracted with TRIzol reagent (Invitrogen, Waltham, MA, USA). First-strand cDNA was synthesized using the PrimeScript™ RT Reagent Kit with gDNA Eraser (Takara, Kyoto, Japan). qRT-PCR was performed on an ABI real-time PCR system (Applied Biosystems, Waltham, MA, USA) with Vazyme qPCR Mix (Vazyme, Nanjing, China) to quantify mRNA levels of: Ecdysteroid (20-hydroxyecdysone, 20E) signaling genes (*EcR*, *USP*, *E74*, *E75B*, *E93*, *BR-C*, *βFtz-F1*), juvenile hormone (JH)-related genes (*Met1*, *Kr-h1*), cell cycle regulators (*Cyclin D*, *Cyclin E*), and silk protein genes (*Fib-H*, *Fib-L*, *P25, Sericin1*, *Sericin3*). Each 30 µL reaction contained 15 µL qPCR Mix, 0.5 µL each of forward and reverse primers, 2 µL cDNA template, and 12 µL ddH_2_O. The thermal cycling conditions were: 94 °C for 20 s, then 40 cycles of 94 °C for 20 s and 60 °C for 20 s. GAPDH served as the internal control [20]. Primer sequences are listed in Table S1. Relative expression was calculated via the 2^–ΔΔCt^ method, with three technical replicates per biological replicate.

### 2.6. Comparison of Economic Characteristics

At 7 d after cocooning, cocooning rate and silk yield were assessed. Cocooning rate (%) was calculated as [(number of cocoons/number of larvae) × 100%]. For silk yield, 150 female and 150 male cocoons were randomly selected per group. The cocoon weight and cocoon shell weight were recorded, and the cocoon shell ratio (%) was calculated as [(cocoon shell weight/cocoon weight) × 100%].

### 2.7. Statistical Analysis

Data analysis and figure preparation were performed using GraphPad Prism 8. Comparisons between two groups employed two-tailed, unpaired Student’s *t*-tests. Significance levels were defined as* *p* < 0.05, ** *p* < 0.01, *** *p* < 0.001, **** *p* < 0.0001.

## 3. Results

### 3.1. Effects of Dietary NaB on Silkworm Growth

We first conducted a dose–response study to assess how different concentrations of NaB affect body growth. As shown in Figure S1, larvae and pupae in the 10 mM NaB group (NaB10) exhibited significantly higher body weights at the onset of wandering and at pupation compared with the water-soaked control (Control), whereas 80 mM NaB (NaB80) treatment caused a marked decrease in both larval and pupal weights. Based on these results, 10 mM was selected for all subsequent experiments.

**Figure 1:**
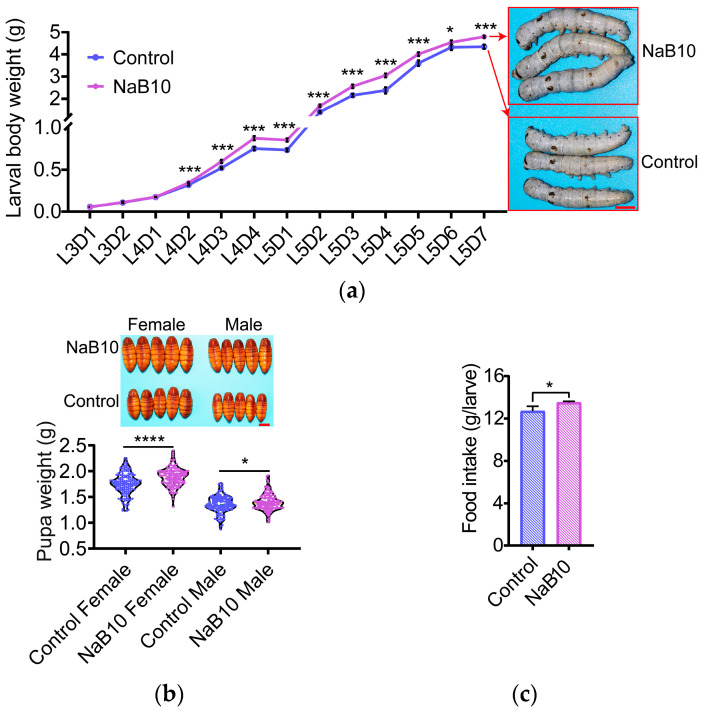
Effects of 10 mM NaB treatment on silkworm growth. (**a**) NaB (10 mM) enhanced larval body weight gain starting at L4D2. Scale bar = 1 cm; (**b**) NaB (10 mM) significantly increased pupal weight (*n* = 150). Scale bar = 1 cm; (**c**) NaB (10 mM) significantly increased total mulberry leaf consumption from L5D1 to L5D7. Data were expressed as mean ± SD. Differences in data were assessed by Student’s *t*-test (two-tailed, two-sample equal variance hypothesis). Significant differences were defined as * *p* < 0.05, *** *p* < 0.001,**** *p* < 0.0001.

Next, we examined the temporal effects of 10 mM NaB treatment on larval growth. Although there was no significant difference in body weight at L3D1 between NaB10 and Control, NaB10 larvae began to outperform Control from L4D2 onward (Figure 1a, *p* < 0.05). By L5D7, larval body weight in the NaB10 group was 10.37% greater than Control (Figure 1a, *p* < 0.05). Correspondingly, pupal weights increased by 7.35% in females and 3.09% in males under NaB10 (Figure 1b; female, *p* < 0.0001; male, *p* < 0.05). These findings indicate that continuous 10 mM NaB treatment exerts a sustained growth-promoting effect.

Finally, total mulberry intake over L5D1–L5D7 was significantly higher in the NaB10 group (Figure 1c, *p* < 0.05), suggesting that enhanced feeding may underlie the observed gains in larval growth and subsequent pupal development.

### 3.2. Effects of NaB on Hormonal Regulatory Pathways in Silkworms

**Figure 2:**
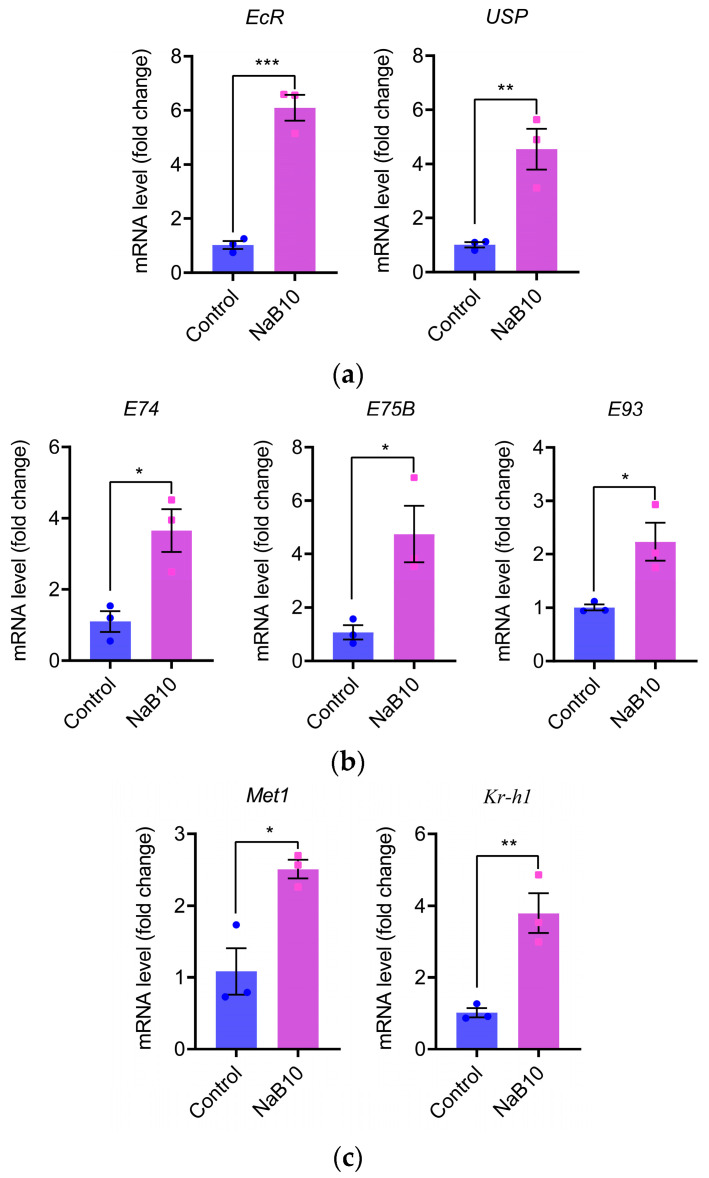
NaB (10 mM) treatment alters hormone signaling pathways in silkworm larvae. Each dot or square represents the mRNA expression level of a specific gene in an individual qPCR sample. (**a**) Upregulation of the 20-hydroxyecdysone receptor genes *EcR* and *USP* following 10 mM NaB treatment; (**b**) increased expression of the 20E-inducible genes *E74*, *E75B*, and *E93* in NaB-supplemented larvae; (**c**) enhanced expression of the juvenile hormone receptor gene *Met1* and the early JH-responsive gene *Kr-h1* after NaB supplementation. The experiments were carried out in three biological repetitions and three mechanical repetitions. The error bars indicate the mean ± SEM, * *p* < 0.05, ** *p* < 0.01, *** *p* < 0.001.

The 20E and JH act synergistically to regulate insect growth and development [21]. To determine whether 10 mM NaB affects these pathways in silkworm, we quantified the expression of key 20E and JH signaling genes in L5D3 larvae by qRT-PCR. As shown in Figure 2a, NaB treatment significantly upregulated the 20E receptor genes *EcR* (*p* < 0.001) and *USP* (*p* < 0.01). Downstream 20E-responsive genes *E74*, *E75B*, and *E93* were also markedly induced (Figure 2b; each *p* < 0.05). Likewise, the JH receptor gene *Met1* and the early JH-response gene *Kr-h1* exhibited a similar pattern of significant upregulation in the NaB10 group (Figure 2c; *Met1 p* < 0.05, *Kr-h1 p* < 0.01). Moreover, 10 mM NaB treatment extended fifth-instar duration (Figure S2). These data demonstrate that 10 mM NaB alters both 20E and JH signaling pathways, suggesting their involvement in NaB-mediated growth regulation.

### 3.3. Effects of Dietary NaB on Silk Gland Development

**Figure 3:**
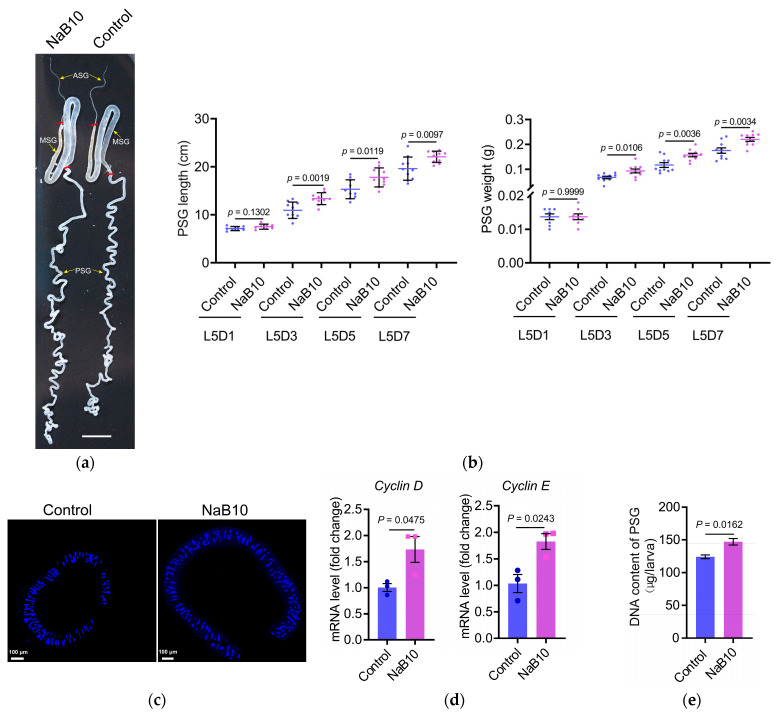
The 10 mM NaB treatment enhances silk gland development. (**a**) Silk gland morphology in L5D7 larvae. Red lines delineate the boundaries between the middle silk gland (MSG) and the anterior (ASG)/posterior (PSG) regions. Scale bar = 1 cm. (**b**) NaB (10 mM) treatment increased PSG length and weight in L5D3–L5D7 larvae (*n* = 10 per time point; *p* < 0.05). Individual points represent measurements of posterior silk gland length or weight from single biological samples. Blue: control group; purple: NaB10 treatment group; (**c**) PSG diameter at L5D7 was visibly greater following NaB supplementation. Scale bars = 100 μm; (**d**) expression of *Cyclin D* and *Cyclin E* in L5D3 PSG was upregulated by NaB treatment (*n* = 3; *p* < 0.05); (**e**) genomic DNA content of L5D7 PSGs was significantly increased in the NaB10 group (n = 3; *p* < 0.05). The error bars indicate the mean ± SEM.

Silk glands from NaB10-treated L5D7 larvae showed significant enlargement versus controls (Figure 3a), with 10.19% length increase and 21.38% weight gain (both *p* < 0.01; Figure S3). The middle silk gland (MSG) displayed similar hypertrophy (Figure S3).

Given that PSG-synthesized fibroins comprise ~75% of cocoon proteins [20], we therefore focused on morphological and molecular changes in the PSG of fifth-instar larvae. As shown in Figure 3b, NaB10 larvae exhibited significantly greater PSG length and weight at L5D3, L5D5, and L5D7 compared with controls (*p* < 0.05). Fluorescence microscopy of L5D7 glands further revealed an increase in gland diameter following NaB treatment (Figure 3c). Together, these data demonstrate that dietary NaB supplementation promotes PSG growth during the mid-to-late fifth instar.

Silk gland enlargement in silkworm larvae is driven by endoreplication—successive genome duplications without cell division—within glandular cells [22]. To determine whether NaB-induced PSG enlargement correlates with enhanced endoreplication, we measured transcript levels of the G/S–phase cyclins Cyclin D and Cyclin E in L5D3 PSG by qRT-PCR. Both cyclins genes were upregulated in the NaB10 group (*Cyclin D p* = 0.0475; *Cyclin E p* = 0.0243; Figure 3d). Consistent with increased cyclin genes expression, total genomic DNA content in NaB-treated PSGs was significantly higher than in controls (*p* = 0.0162; Figure 3e). These findings indicate that 10 mM NaB treatment promotes DNA replication during endoreplication cycles, thereby driving PSG cell growth and overall gland enlargement.

### 3.4. Effects of Dietary NaB on Economic Traits

**Figure 4:**
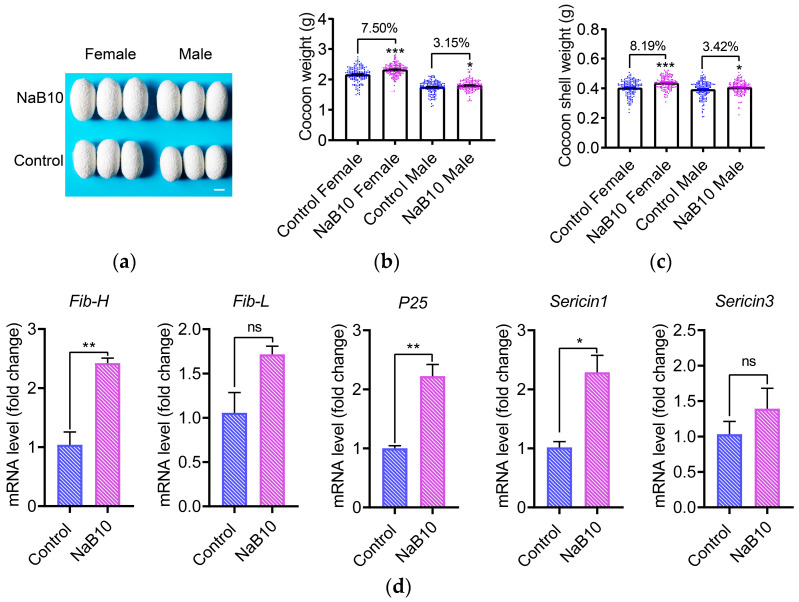
Effects of 10 mM NaB on economic traits of silkworm. (**a**) Representative cocoon morphology demonstrating size enlargement under NaB10 treatment. Scale bar = 1 cm; (**b**) 10 mM NaB significantly increased cocoon weight; (**c**) 10 mM NaB significantly increased cocoon shell weight; (**d**) RT-qPCR analysis of silk protein genes expression (*n* = 3). Error bars represent mean ± SEM. Significance levels: * *p* < 0.05, ** *p* < 0.01, *** *p* < 0.001. “ns” indicates non-significant difference.

To evaluate the impact of 10 mM NaB treatment on economically important cocoon traits, we first examined cocoon morphology. Larvae receiving NaB supplementation produced visibly larger cocoons compared to the control group (Figure 4a). Quantitative assessment revealed that NaB treatment increased cocoon weight by 7.50% in females (*p* < 0.001) and 3.15% in males (*p* < 0.05) (Figure 4b). Cocoon shell weight was similarly elevated by 8.19% in females (*p* < 0.001) and 3.42% in males (*p* < 0.05) (Figure 4c). Furthermore, NaB treatment significantly upregulated transcription of silk protein-coding genes *Fib-H*, *P25*, and *Sericin1* (*p* < 0.05; Figure 4d). Although the cocoon shell ratio and cocooning rate remained unaffected (*p* > 0.05; Figure S4), these data demonstrate that optimal NaB supplementation can significantly improve silk yield in the silkworm without impairing overall cocooning performance.

## 4. Discussion

This study demonstrates that an appropriate NaB supplementation positively regulates silkworm growth, as evidenced by significant increases in both larval and pupal weights compared with controls. These findings are consistent with reports in other livestock species. For example, Li et al. (2019) showed that adding 0.5% NaB to rabbit diets significantly increased both full- and half-carcass weights [23], while Deng et al. (2023) found that supplementing broiler feed with 1000 mg/kg coated sodium butyrate (CSB) for 21 days raised average daily gain by 6.62% and final body weight by 6% [24]. Likewise, Elnesr et al. (2019) reported that 1 g/kg NaB in the diet of *Coturnix coturnix japonica* increased body weight at day 21 [25], and Hou et al. (2023) observed that 2.0 g/kg NaB in *Micropterus salmoides* feed significantly boosted weight at 56 days [26]. Together, these studies suggest a broadly conserved growth-promoting effect of NaB across animal taxa.

However, the efficacy of NaB often depends on dose [13,27]. Lan et al. (2020) reported that 0.6 g/kg NaB improved broiler ADG, whereas 1.2 g/kg actually inhibited growth, and 0.3 g/kg had no effect [28]. Similarly, Wu et al. (2023) identified an optimal 8.78 g/day NaB dose in pre-weaning calves, with 17.6 g/day slightly reducing ADG [14], and Akram et al. (2024) showed that injecting 0.3% NaB into chicken eggs increased post-hatch weight, whereas 0.5% lowered it [29]. In line with these reports, we observed that low concentrations of NaB (2.5–5 mM) had no significant effect on silkworm weight, while higher concentrations (20–80 mM) adversely affected larvae or pupae (Figure S1). The dose-dependence likely due to NaB’s rapid metabolism and absorption in the gut: most ingested salt is consumed before reaching target tissues [13]. Dai et al. (2023) demonstrated that microencapsulated NaB more effectively promoted *Gallus gallus domesticus* intestinal development than uncoated NaB, which is degraded prematurely in the upper gut [30]. In ruminants, excessive NaB (17.6 g/day) reduced rumen microbial diversity and beneficial taxa [14,31], and Qiu et al. (2017) showed high NaB concentrations can induce apoptosis in porcine jejunal epithelial cells [32]. Moreover, the characteristic odor of NaB may deter feed intake at high doses [14,30]. Together, these findings indicate that NaB’s growth-promoting actions require a narrow concentration window to balance effects on gut microbiota, cell viability, and palatability. Future studies should investigate NaB’s effects on silkworm midgut development, microbial symbionts, and feeding behavior to elucidate the mechanisms underlying its dose-dependent efficacy.

In this study, continuous 10 mM NaB treatment from the third through fifth instars modulated body growth, particularly in the fourth and fifth instars. Notably, while control larvae reached maximum weight by L5D6, NaB-treated larvae continued to gain weight during L5D6–L5D7. Pupal weight was likewise significantly increased in the NaB10 group. These results contrast with those of Wu et al. (2023) and Lan et al. (2020), who reported that although NaB supplementation enhanced early growth performance in pre-weaning calves and broilers, it had no significant effect on average daily gain or final body weight in later stages [14,28]. We propose that the divergent outcomes arise from fundamental differences in insect versus vertebrate development. As a holometabolous insect, silkworm undergoes a rapid “feeding boom” during the fourth and fifth instars to accumulate reserves for metamorphosis, rendering its gut metabolism more responsive to exogenous NaB. In contrast, mammals and birds typically approach metabolic homeostasis in their later growth phases, reducing sensitivity to continued NaB supplementation.

The 10 mM NaB treatment significantly increased mulberry leaf intake by silkworm larvae during L5D1–L5D7. Similarly, Liu et al. (2019) reported that oral gavage of 0.36 g/kg NaB in neonatal lambs significantly elevated average daily feed intake from days 10 to 49 [33]. Upadhaya et al. (2020) further demonstrated that inclusion of coated sodium butyrate in weaned pig diets increased feed intake over days 1–42 [34]. In contrast, Wu et al. (2023) and Dahiya et al. (2016) found no significant effect of NaB on feed intake in calves or laying hens [14,35]. Such discrepancies likely reflect differences in species, developmental stage, NaB dose, or administration method. NaB has been shown to enhance ruminal papilla growth in lambs [33], and dietary NaB can increase digestive enzyme activities in *Pelteobagrus fulvidraco* [36]. In poultry, NaB supplementation has been reported to increase the relative weight and length of the duodenum, jejunum, and ileum [28], as well as jejunal villus height in chickens, piglets, and lambs, thereby expanding the absorptive surface area [34,37,38]. We therefore hypothesize that an appropriate dose of dietary NaB not only stimulates *Bombyx mori* larval feeding but also enhances gut development and nutrient absorption, collectively providing additional energy for somatic growth.

In this study, treatment with 10 mM NaB simultaneously upregulated the expression of 20E signaling pathway genes (*EcR*, *USP*, *E74*, *E75B* and *E93*) and key JH pathway genes (*Met1* and *Kr-h1*) in mid-fifth-instar larvae. Two potential regulatory mechanisms may underlie these effects. First, ample nutrient availability (e.g., amino acids, fatty acids and carbohydrates) is known to activate the target of rapamycin (TOR) pathway in the prothoracic gland, thereby promoting 20E biosynthesis [38,39]. Accordingly, the NaB-induced increase in larval feeding may enhance 20E synthesis and thus drive transcription of 20E-responsive genes; future quantification of hemolymph 20E titers by high-performance liquid chromatography would provide direct validation. More importantly, NaB’s epigenetic regulatory activity as a class I and IIa histone deacetylase (HDAC) inhibitor may directly modulate transcription of both JH and 20E pathway genes. By increasing histone acetylation, NaB relaxes chromatin structure, enhances promoter accessibility to transcription factors, and reverses HDAC-mediated transcriptional repression [40,41,42]. Indeed, HDACs have been implicated in regulation of JH- and 20E-related gene expression in *Tribolium castaneum* [43,44]: RNAi-mediated knockdown of *HDAC1* in late-instar larvae elevates expression of the early JH-response gene *Kr-h1*, the JH-induced Rho GTPase-activating protein 100F (LOC660562) and lachesin (LOC659929) [43], while treatment of *Tribolium castaneum* cells with the HDAC inhibitor trichostatin A (TSA) simultaneously induces *Kr-h1* and 20E pathway genes *Ftz-F1*, *HR51* and *SVP* [44]. Similarly, knockdown of *HDAC1*, *HDAC4* and *HDAC11* in *Aedes aegypti* larvae promotes *Kr-h1* expression and delays larval–pupal transition [45], and RNAi against *HDAC3* in *Helicoverpa armigera* alters JH and 20E pathway gene profiles, upregulating *Kr-h1* as well as the 20E-induced factors *BR-C* and *E78* [46]. Notably, although NaB activated early 20E-response genes, downstream metamorphic programs were not prematurely triggered: expression of the pupal metamorphosis regulator *βFtz-F1* remained unchanged (Figure S5), and the larval–pupal transition was delayed (Figure S2), indicating that JH pathway activity continues to dominate development under NaB treatment. These findings enrich the growing evidence that HDACs, via epigenetic regulation, participate in insect growth and development. Future studies employing acetyl-proteome quantitative mass spectrometry could map NaB-induced histone acetylation patterns and identify specific HDAC target proteins.

Silk glands are the sole organs in *Bombyx mori* responsible for the synthesis and secretion of silk proteins, and their development is tightly linked to cocoon silk yield [1]. Previous work has shown that supplementation with growth regulators can enhance silk gland development in silkworm larvae [10]. In the present study, 10 mM NaB treatment significantly increased silk gland length, weight, and the diameter of PSG, demonstrating that an appropriate NaB dose promotes silk gland growth.

At the cellular level, silk gland enlargement in larval *Bombyx mori* depends on endoreplication—repeated rounds of G and S phases without mitosis—leading to increased cell size and gland volume [22,47]. *Cyclin D* and *Cyclin E* are key regulators of the G/S transition in endocycling cells [47]. We found that 10 mM NaB treatment upregulated *Cyclin D* and *Cyclin E* expression in PSG cells and concurrently elevated total PSG DNA content. These molecular changes mirror observations in other species: Liu et al. (2019) reported that exogenous NaB significantly upregulated *Cyclin A2* and *Cyclin D1* in lamb rumen epithelium [33], and Zhang et al. (2023) showed that NaB increased *Cyclin A2* and *Cyclin D1* mRNA levels, promoting bovine mammary epithelial cell proliferation [48]. We therefore propose that NaB drives PSG development by enhancing endoreplication-associated DNA synthesis, thereby providing the cellular capacity for massive fibroin production in the late fifth instar.

In line with the enhanced silk-gland development, treatment with 10 mM NaB significantly increased cocoon-shell weight and upregulated the expression of Fib-H, P25, and Sericin1 genes, indicating that silk protein synthesis was promoted. Similar positive effects of NaB on protein production have been documented in mammals: dietary NaB increased milk and milk protein content in dairy cows [48], and supplementation of 800 mg/kg NaB improved laying rate in hens [19]. To identify practical NaB dosages for sericulture, we evaluated cocoon traits under low (2.5–5 mM) and high (80 mM) NaB concentrations. Low doses had no effect, whereas 80 mM NaB reduced silk yield (Figure S6). Importantly, 10 mM NaB did not compromise cocooning rate, suggesting that this dosage can be safely applied in sericulture to boost silk output. Finally, we observed a sex-specific response to NaB: shell weight increased by 8.19% in females versus 3.42% in males under 10 mM NaB. This raises the prospect that tailored NaB supplementation in female-only rearing systems may further maximize silk production.

Xiao et al. (2023) reported that dietary supplementation with NaB significantly enhanced laying performance, egg quality, and hatchability in laying hens [49]. Another study demonstrated that NaB increased semen volume, improved sperm motility, and reduced the proportion of abnormal sperm in roosters, thereby boosting male reproductive performance [50]. In the present study, dietary NaB supplementation concurrently increased pupal weight in both female and male silkworm, suggesting that NaB may also hold promise for seed-cocoon production aimed at egg harvest.

## 5. Conclusions

This study demonstrates that dietary supplementation with an appropriate concentration of NaB promotes silkworm body growth and silk gland development, resulting in significant improvements in key economic traits. Mechanistically, NaB appears to act by enhancing nutrient intake, effecting the juvenile hormone and ecdysteroid signaling pathways, and stimulating endoreplication-associated DNA synthesis in silk gland cells. Our findings broaden the potential applications of short-chain fatty acid salts in sericulture and offer novel insights for strategies aimed at overcoming current bottlenecks in silk yield enhancement.

## Data Availability

The original contributions presented in this study are included in the article/Supplementary Materials. Further inquiries can be directed to the corresponding authors.

## Supplementary Materials

The following supporting information can be downloaded at: https://www.mdpi.com/article/10.3390/insects16080761/s1, Table S1: primers used in the present study; Figure S1: Effects of different concentrations of NaB treatment on silkworm growth; Figure S2: treatment with 10 mM NaB prolonged the L5 stage in silkworms; Figure S3: the silk gland (SG) and its middle region (MSG) showed significant enlargement in L5D7 larvae following 10 mM NaB treatment; Figure S4: 10 mM NaB treatment had no significant effect on cocoon shell ratio or cocooning rate; Figure S5: qPCR analysis of *βFtz-F1* mRNA levels in third-day fifth-instar larvae following 10 mM NaB treatment; Figure S6: effects of dietary supplementation with different NaB concentrations on silkworm economic traits. Reference [51] is cited in the supplementary materials.

## Abbreviations

The following abbreviations are used in this manuscript:

NaB	Sodium butyrate
SG	Silk gland
MSG	Middle silk gland
PSG	Posterior silk gland
