# Supplementary material for: Dietary Sodium Butyrate Supplementation Enhances Silkworm Silk Yield by Simultaneously Promoting Larval Growth and Silk Gland Development
Source: Insects. 2025 Jul 24;16(8):761. doi: 10.3390/insects16080761 (PMC12386993; doi:10.3390/insects16080761)
Supplement: Supplementary file 1 [file insects-16-00761-s001.zip › insects-3736179-supplementary.pdf]

Supplementary Materials

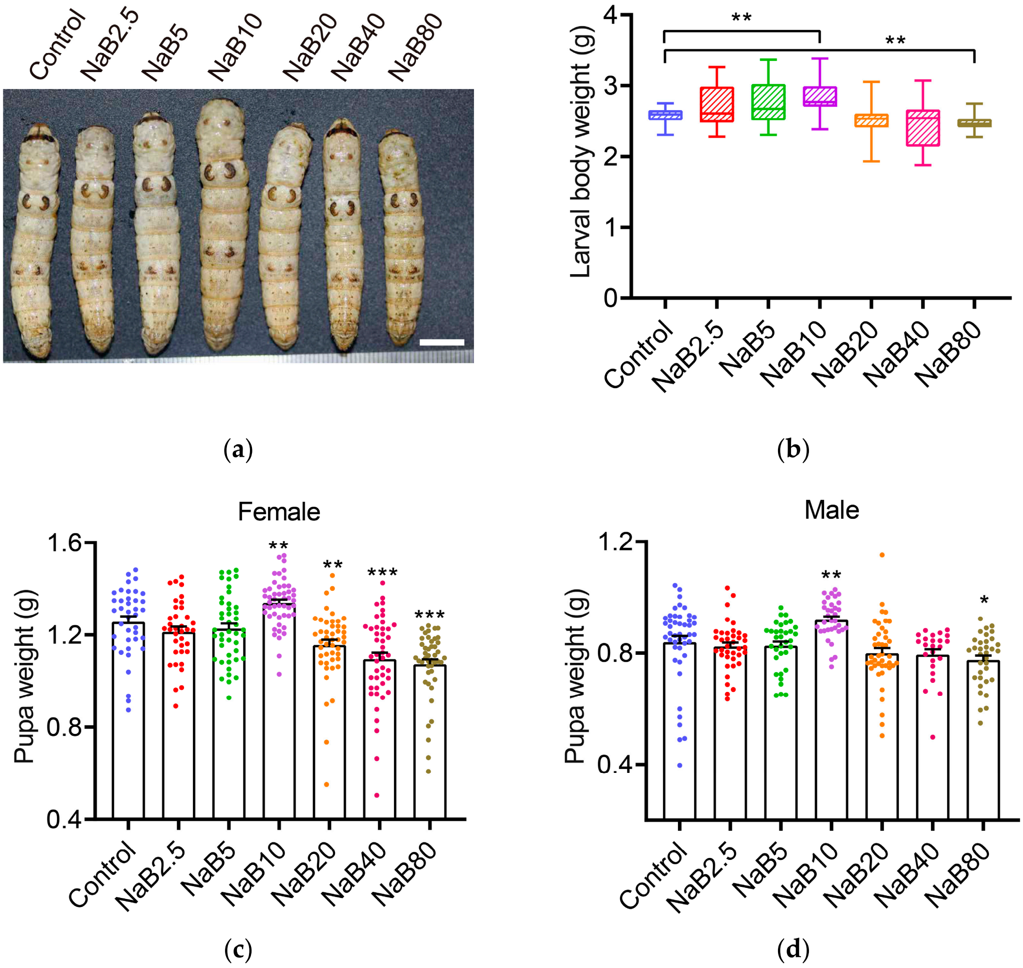

Figure S1. Effects of different concentrations of NaB treatment on silkworm growth. (a) Morphological observations of larvae at the early wandering stage following supplementation with different concentrations of NaB. Scale bar: 1 cm. (b) Larval body weight analysis at the wandering stage (n = 18). (c-d) Pupal weight analysis across NaB concentrations. Each data point represents measurements from an individual biological sample. (Female: n = 39, 36, 44, 49, 46, 45, 45; Male: n = 45, 38, 34, 34, 42, 24, 34). Data were expressed as mean ± SEM. Differences in data were assessed by Student's t-test. Significant differences were defined as * $p < 0.05$, ** $p < 0.01$, *** $p < 0.001$.

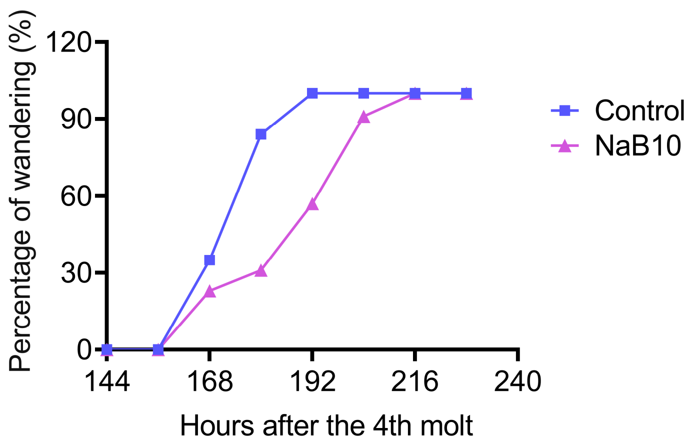

Figure S2. Treatment with 10 mM NaB prolonged the L5 stage in silkworms.

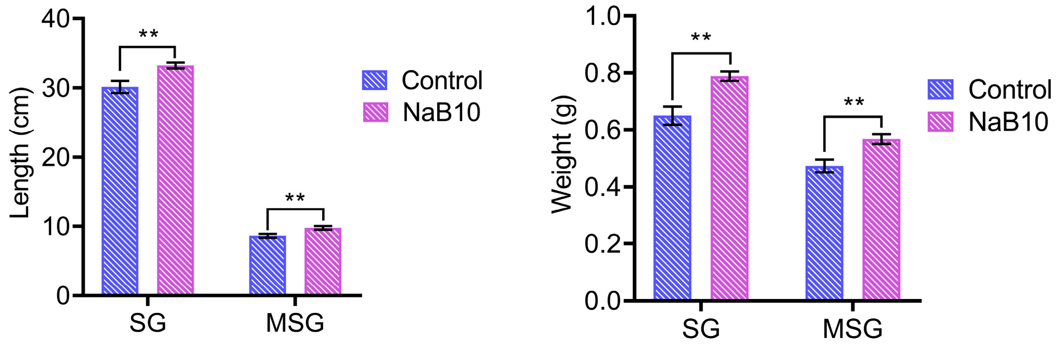

(**a**) (**b**)

Figure S3. Treatment with 10 mM NaB significantly increased the length (a) and weight (b) of both silk gland (SG) and its middle region (MSG). Data were expressed as mean ± SEM. Significant differences were defined as ** $p < 0.01$.

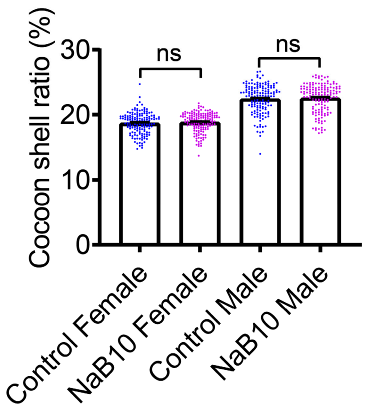

(**a**)

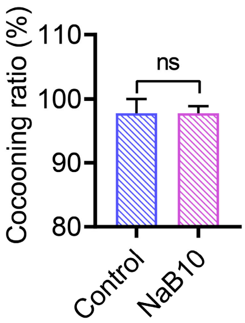

(**b**)

Figure S4. 10 mM NaB treatment had no significant effect on cocoon shell ratio (a) or cocooning rate (b). Each data point represents measurements from an individual biological sample. Data were expressed as mean ± SEM. "ns" indicates non-significant difference.

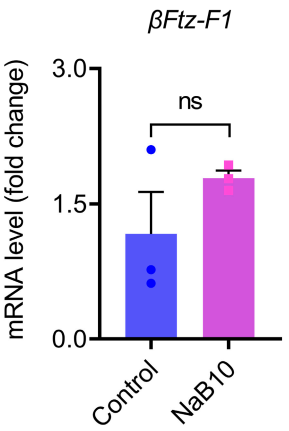

Figure S5. qPCR analysis of *βFtz-F1* mRNA levels in third-day fifth-instar larvae following 10 mM NaB treatment. Each dot or square represents the mRNA expression level of a specific gene in an individual qPCR sample. “ns” denotes no significant difference.

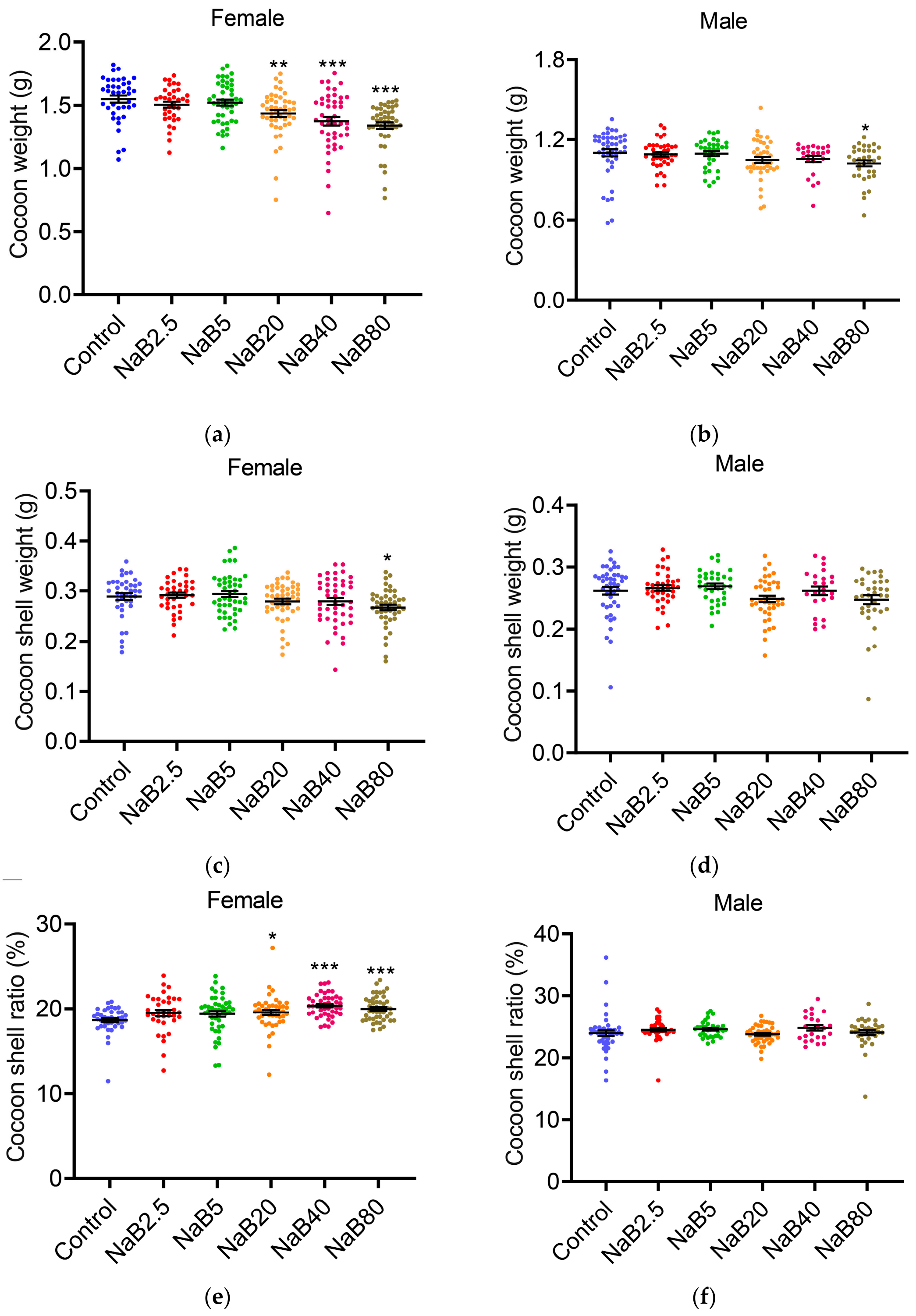

Figure S6. Effects of dietary supplementation with different NaB concentrations on silkworm economic traits. Each data point represents measurements from an individual biological sample. Sample sizes: female n = 39, 36, 44, 49, 46, 45, 45; male n = 45, 38, 34, 34, 42, 24, 34). (a,b) Cocoon weight was significantly decreased in females at 20-40 mM ($p < 0.01$), and in both sexes at 80 mM (female $p < 0.001$; male $p < 0.05$). (c,d) Cocoon shell weight showed significant reduction only in 80 mM-treated females ($p < 0.05$). (e,f) Cocoon shell ratio was significantly increased in females across 20-80 mM range. Data were expressed as mean ± SEM. Differences in data were assessed by Student's t-test. Significant differences were defined as * $p < 0.05$, ** $p < 0.01$, *** $p < 0.001$.

Table S1. Primers used in the present study

Purpose	Primer name	Primer sequence
qRT-PCR	*ECR*	Forward: 5' GGCAGTCGGATGAAGAGGAC 3'<br>Reverse: 5' AAAAGCCCGGTAGACCCTTG 3'
	USP	Forward: 5' CCGTATCGGCCGTGAGTC 3'<br>Reverse: 5' ACGGCCTCCGTAAAGATAGC 3'
	Met1	Forward: 5' AAAACTCGTCGCAGCCAGTA 3'<br>Reverse: 5' AGACGTGTTCGTTACGCAGT 3'
	Kr-h1	Forward: 5' TCCCAGTATTTCAGTGGCTTCA 3'<br>Reverse: 5' ATGTAATGTACGGGGACCGC 3'
	Br-C	Forward: 5' GCTCCACTGATAACTCGCCT 3'<br>Reverse: 5' ACCCCTGCTGTTCAGAATCG 3'
	E74	Forward: 5' GACAGTTTCAGGACGCGGTA 3'<br>Reverse: 5' AGTTCAACAACGGAAGAAAGTC 3'
	E75B	Forward: 5' GGGGCGCGAAGATTAGAACT 3'<br>Reverse: 5' ATGGAGCTCGACGGTTCTTC 3'
	E93	Forward: 5' CGATGCCGAGCAATTCCAAG 3'<br>Reverse: 5' ACATCTCTCCTCGCTGGACT 3'
	βFtz-F1	Forward: 5' CCACAACGGGCAGAAGTTTG 3'<br>Reverse: 5' CACGAACGCACTTCACGTTT 3'
	CyclinD[47]	Forward: 5' GCGTTTGACTTCGTGGAACC 3'<br>Reverse: 5' CGTTGACAGACATTCGCACG 3'
	CyclinE[47]	Forward: 5' CCCAAGACAATCCAGGCAA 3'<br>Reverse: 5' AGAGGCGAGTCCACCCCA 3'
	Fib-H	Forward: 5' TTGTGATCTTGTGCTGCGC 3'<br>Reverse: 5' CAATGGACTCGTTACCGTCG 3'
	Fib-L	Forward: 5' TCTCGGTCCCTTCTTCG 3'<br>Reverse: 5' GTTGTTGCTTTGGCTGTT 3'
	P25	Forward: 5' GTCTGCCCATCTTCCACA 3'<br>Reverse: 5' GCCAGTTCCTCTTTCCG 3'
	Sericin1	Forward: 5' ACAGTTACGACAAGGGCTA 3'<br>Reverse: 5' TCCATCTGAAGTTTCTACGAC 3'
	Sericin3[51]	Forward: 5′-AGTTGCTCTATTCCTGATAG-3′<br>Reverse: 5′-TGTCGTCGGAATTCTCACCA-3′
